# Oxidized Low-Density Lipoprotein Associates with Ventricular Stress in Young Adults and Triggers Intracellular Ca^2+^ Alterations in Adult Ventricular Cardiomyocytes

**DOI:** 10.3390/antiox9121213

**Published:** 2020-12-01

**Authors:** Elena Rodríguez-Sánchez, José Alberto Navarro-García, Laura González-Lafuente, Jennifer Aceves-Ripoll, Sara Vázquez-Sánchez, Jonay Poveda, Elisa Mercado-García, Nerea Corbacho-Alonso, Eva Calvo-Bonacho, María Fernández-Velasco, Gloria Álvarez-Llamas, María G. Barderas, Luis M. Ruilope, Gema Ruiz-Hurtado

**Affiliations:** 1 Cardiorenal Translational Laboratory, Institute of Research i+12, Hospital Universitario 12 de Octubre, 28041 Madrid, Spain; erodsanchez.imas12@h12o.es (E.R.-S.); jalbertong.imas12@h12o.es (J.A.N.-G.); lgonzlafuente.imas12@h12o.es (L.G.-L.); jen.ace.rip@hotmail.com (J.A.-R.); sara.vazquez.imas12@h12o.es (S.V.-S.); jpoveda.imas12@h12o.es (J.P.); elisamercado21@gmail.com (E.M.-G.); ruilope@icloud.com (L.M.R.); 2 Department of Vascular Physiopathology, Hospital Nacional de Parapléjicos, SESCAM, 45004 Toledo, Spain; nerea_ca_@hotmail.com (N.C.-A.); megonzalezb@sescam.jccm.es (M.G.B.); 3 IBERMUTUA, 28043 Madrid, Spain; evacalvo@ibermutua.es; 4 IdiPAZ Institute for Health Research/Centro de Investigación Biomédica en Red de Enfermedades Cardiovasculares, CIBER-CV, 28029 Madrid, Spain; mvelasco@iib.uam.es; 5 Departament of Immunology, IIS-Fundación Jimenez Diaz, 28040 Madrid, Spain; GAlvarez@quironsalud.es; 6 Hypertension Unit, Hospital Universitario 12 de Octubre, 28041 Madrid, Spain; 7 CIBER-CV, Hospital Universitario 12 de Octubre, 28041 Madrid, Spain; 8 European University of Madrid, Madrid, Spain

**Keywords:** oxidized LDL, NT-proBNP, native adult ventricular cardiomyocytes, Ca^2+^ handling

## Abstract

Oxidized low-density lipoprotein (oxLDL) is associated with cardiac damage and causes injury to multiple cell types. We aimed to investigate the role of oxLDL in ventricular stress. We first examined the association between circulating oxLDL and N-terminal pro-brain natriuretic peptide (NT-proBNP), a marker of myocardial stress, in young subjects (30–50 years) with or without stable coronary artery disease (SCAD). oxLDL and NT-proBNP were significantly higher in subjects at high cardiovascular risk (CVR) than in subjects at low CVR and were associated independently of traditional CVR factors and C-reactive protein. Furthermore, the levels of oxLDL and NT-proBNP were significantly lower in subjects with SCAD than in peers at high CVR. To determine the intracellular mechanisms involved in the cardiac effects of oxLDL, we analyzed the in vitro effect of oxLDL on intracellular Ca^2+^ handling in adult rat ventricular cardiomyocytes using confocal microscopy. Acute challenge of adult ventricular cardiomyocytes to oxLDL reduced systolic Ca^2+^ transients and sarcoplasmic reticulum Ca^2+^ load. Moreover, diastolic spontaneous Ca^2+^ leak increased significantly after acute exposure to oxLDL. Thus, we demonstrate that oxLDL associates with NT-proBNP in young subjects, and can directly induce Ca^2+^ mishandling in adult ventricular cardiomyoyctes, predisposing cardiomyocytes to cardiac dysfunction and arrhythmogenicity.

## 1. Introduction

Cardiovascular disease is the leading cause of death and disability worldwide. Among cardiovascular risk (CVR) factors, one of the major contributors to atherosclerosis development and progression is raised serum levels of low-density lipoprotein (LDL) cholesterol, which can penetrate the vascular wall and accumulate in the subendothelial space. LDL is highly susceptible to oxidation in the environment of the atheromatous plaque. Oxidized LDL (oxLDL) exacerbates the atherosclerosis process by triggering pro-oxidant and pro-inflammatory pathways that lead to plaque instability through mechanisms independent of LDL [1,2]. Consequently, oxLDL is causally associated with the incidence of coronary events even in the setting of moderate CVR [3,4]. However less is known about the intracellular mechanisms by which oxLDL induces deleterious cardiac effects, especially at the level of cardiomyocytes.

Cardiomyocyte electrical activity and contractility are governed by excitation–contraction (EC) coupling, which is the physiological process of converting an electrical signal into mechanical work. Ca^2+^ represents a key player in cardiac EC coupling [5]. During EC coupling, the action potential depolarizes the membrane, opening L-type Ca^2+^ channels at the sarcolemma and allowing a small influx of Ca^2+^ into the cytoplasm. This increase in cytoplasmic Ca^2+^ concentration induces the activation of ryanodine receptors at the sarcoplasmic reticulum (SR) and the subsequent release of Ca^2+^ from the SR to the cytoplasmic space, elevating the intracellular concentration of Ca^2+^, which binds to myofilaments and activates contraction [6]. During relaxation, cytosolic Ca^2+^ is pumped back to the SR mainly by the SR-Ca^2+^-adenosine triphosphatase 2a (SERCA) pump and, to a lesser extent, is extruded outside of the cell by the Na^+^-Ca^2+^ exchanger (NCX) [6]. Alterations in EC coupling and Ca^2+^ handling are related to contractile dysfunction and arrhythmias [7]. Previous studies on the effect of oxLDL at the level of cardiomyocytes focused on its sustained effect [8,9,10]. However, the effects of acute alterations in oxLDL levels are also relevant, as it is well-known that oxLDL induces the expression of inflammatory mediators and reactive oxygen species (ROS), which in turn might affect cardiomyocyte function [7,11].

The aim of the present study was to question whether oxLDL is associated with cardiac dysfunction at an early age and before the onset of overt cardiovascular disease. To do this this, we investigated the association between oxLDL and N-terminal pro-B-type natriuretic peptide (NT-proBNP), a marker of ventricular stress, in a young population with or without previous coronary events. At the cellular level, we assessed in vitro the acute effect of oxLDL on Ca^2+^ handling in adult rat ventricular cardiomyocytes.

## 2. Materials and Methods

### 2.1. Human Study

In this cross-sectional study, 66 subjects aged 30–50 years were recruited from Ibermutua [12], a Spanish insurance company for occupational accidents and disease, during their annual medical examination. Those subjects with a recent cardiovascular event (myocardial infarction or angina in the last 3 years) that was stable and well-controlled on medical therapy were classified as having stable coronary artery disease (SCAD) (N = 23). The remaining participants were classified according to their estimated lifetime CVR using the QRisk-lifetime estimator—including age, sex, ethnicity, diabetes mellitus, family history of angina or heart attack, chronic kidney disease, blood pressure treatment, atrial fibrillation, rheumatoid arthritis, smoking status, cholesterol/high-density lipoprotein ratio, systolic blood pressure and BMI [13]—into those with low-lifetime CVR (<26%, N = 22) or high-lifetime CVR (>26%, N = 21) [14].

All subjects underwent clinical examination and blood analysis. Blood samples were collected in ethylenediamine tetraacetic acid (EDTA) tubes and immediately centrifuged at 2000 rpm for 10 min at 4 °C. Plasma was stored at −80 °C. Plasma levels of NT-proBNP and C-reactive protein (CRP) were determined using commercial ELISA kits (R&D Systems, Minneapolis, MN, USA). Plasma levels of oxLDL were determined with a commercial sandwich ELISA based on the monoclonal antibody 4E6 (Mercodia AB, Uppsala, Sweden), which binds approximately 1000 times stronger to oxLDL than to native LDL [15], in order to minimize cross-reactivity with LDL.

This study was approved by the ethics committee of the Hospital Universitario 12 de Octubre (Madrid), and was performed according to the tenets of the Declaration of Helsinki. All patients signed an informed consent.

### 2.2. Experimental Study

The animal study was conducted following the recommendations of the Spanish Animal Care and Use Committee according to the Guidelines for ethical care and welfare (2013/175) of experimental animals of the European Union (2010/63/EU), and was approved by the General Direction of Agriculture and the Environment at the Environment Council of Madrid (PROEX 053/16).

#### 2.2.1. Cardiomyocyte Isolation

Adult ventricular cardiomyocytes were isolated from hearts of young adult male Wistar rats (150–200 g). Rats were sacrificed with sodium pentobarbital–heparin (intraperitoneal injection, 100 mg/kg–4 UI/g) and hearts were quickly removed and cannulated via the ascending aorta on a Langendorff perfusion system. Hearts were retrograde perfused with calcium-free Tyrode’s solution supplemented with 0.2 mM ethylene glycol tetraacetic acid (EGTA) for 3–7 min at 37 °C, followed by 3–5 min perfusion with Tyrode’s solution supplemented with 0.1 mM CaCl_2_, 1 mg/mL type II collagenase (Worthington, Lakewood, NJ) and 1 mg/mL bovine serum albumin. The left ventricle was then removed, cut into pieces and stirred for 1 min in Tyrode’s solution containing 0.1 mM CaCl_2_. The resultant cell suspension was filtered through a nylon mesh (250 μm) and centrifuged for 3 min at 500 rpm. Cells were resuspended in Tyrode’s solution containing 0.5 mM CaCl_2_, centrifuged again for 3 min at 500 rpm, and finally resuspended in a storage solution containing 1 mM CaCl_2_ [16,17].

#### 2.2.2. Intracellular Ca^2+^ Imaging

Only excitable and Ca^2+^ tolerant rod-shaped cardiomyocytes were included in the study. Ventricular cardiomyocytes were perfused with vehicle solution followed by 3–4 min perfusion with 10 μg/mL oxLDL (#770252, Kalen Biomedical, Montgomery, MD, USA) [8,9,10].

Isolated cardiomyocytes were preloaded with the calcium-sensitive fluorescent dye Fluo-3AM (5 μM; Invitrogen, Carlsbad, CA, USA). Images were obtained with a MetaZeiss LSM 510 confocal microscope (objective w.i. 40×) by tracing a line across the longitudinal axis of the cells. Ca^2+^ transients were recorded in cells field stimulated at 1 Hz using two electrodes for 1 min to reach a steady state. Fluorescence (F) values were normalized to the basal fluorescence (F_0_) to obtain the fluorescence ratio (F/F_0_). The decay time constant of Ca^2+^ transients–Tau–was obtained by fitting the decay time trace to a single exponential. The SR Ca^2+^ load was estimated as the amplitude of caffeine-induced Ca^2+^ transients (F/F_0_) after 10 mM caffeine administration.

Quiescent Fluo-3AM-loaded cells were scanned to record spontaneous Ca^2+^ sparks, which were defined as localized, rapid and brief elevations in Ca^2+^ fluorescence of at least 3 times the standard deviation of F_0_ after Ca^2+^ transient recordings. Pro-arrhythmogenic Ca^2+^ events were assessed by determining the frequency of Ca^2+^ waves, which were identified as increases in fluorescence that start locally and propagate to both sides of the cell. The fluorescence registered was corrected by the background fluorescence in each image.

Confocal Ca^2+^ images were analyzed using home-made routines in Interactive Data Language (IDL; Research Systems, Inc., Boulder, CO, USA) and Image J (NIH, Bethesda, MD, USA).

### 2.3. Statistical Analysis

Normality was assessed with the Kolmogorov–Smirnov test. For the human study, groups were compared using analysis of variance with Newman–Keuls correction or Fisher’s test for categorical variables. Correlations were examined using Pearson’s r correlation coefficient. Multiple linear regression analysis was performed to determine whether the association between oxLDL and NT-proBNP was independent of LDL and CRP levels, sex, body mass index (BMI) and systolic blood pressure. In the animal study, groups were compared using paired Student’s *t*-test for normally distributed variables, or the Mann–Whitney test for not normally distributed variables. Results are expressed as mean±standard error of the mean (SEM), and *p*-values < 0.05 were considered significant. Analyses were performed with GraphPad Prism 6 (GraphPad Software Inc., San Diego, CA, USA), IBM SPSS Statistics v22 (IBM, Armonk, NY, USA), and OriginPro 9.0 (OriginLab, Northampton, MA, USA).

## 3. Results

### 3.1. Study Population

Subjects were stratified into three groups according to whether or not they had SCAD and according to lifetime CVR (Table 1). The mean age of the study subjects was 44 ± 5 years. There were no significant differences between groups in terms of age or renal function parameters. The group classified as low-lifetime CVR had significantly fewer males than the high-lifetime CVR group (*p* < 0.01). BMI was also lower in the low-lifetime CVR group than in the high-lifetime CVR group (*p* < 0.001) and the SCAD group (*p* < 0.01). Hypertension was more prevalent in the high-lifetime CVR and SCAD groups than in the low-lifetime CVR group (*p* < 0.001 and *p* < 0.01, respectively). Values for systolic blood pressure followed the same pattern, but were significantly lower in the SCAD group than in the high-lifetime CVR group (*p* < 0.001). The high-lifetime CVR group had greater levels of total cholesterol, LDL, and triglycerides than the low-lifetime CVR (*p* < 0.05, *p* < 0.001, *p* < 0.001, respectively) or SCAD (*p* < 0.001) groups. Moreover, the SCAD group had lower levels of total cholesterol and LDL than the low-lifetime CVR group (*p* < 0.001, *p* < 0.05), which is likely because all subjects with SCAD were chronically treated with statins. However, high-density lipoprotein levels were significantly higher in the low-lifetime CVR group than in the other groups (*p* < 0.001).

### 3.2. CRP, NT-proBNP and oxLDL Levels in Young Adults

**Figure 1:**
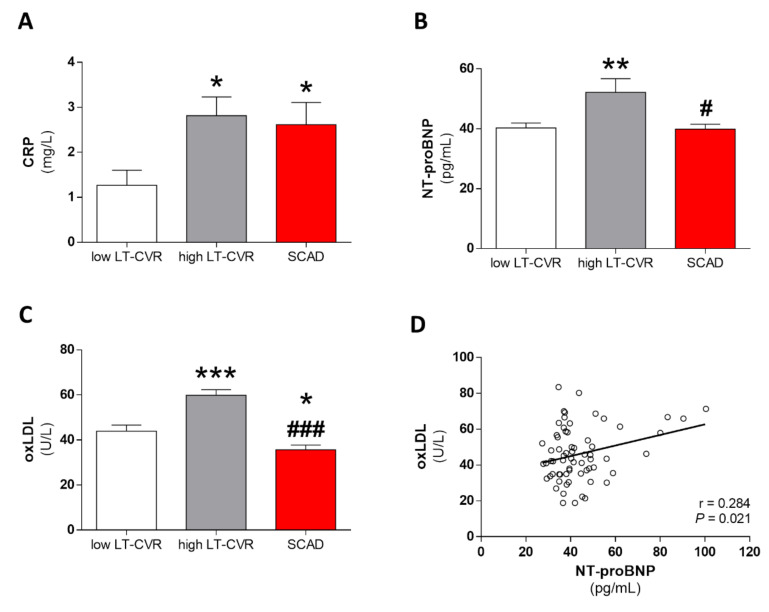
Serum biomarkers in a young population with and without previous cardiovascular disease. Serum levels of biomarkers in subjects with high-lifetime cardiovascular risk (LT-CVR), low-lifetime cardiovascular risk (LT-CVR) or stable coronary artery disease (SCAD). (**A**) C-reactive protein (CRP). (**B**) N-terminal pro-B-type natriuretic peptide (NT-proBNP). (**C**) Oxidized LDL (oxLDL). (**D**) Correlation analysis of oxLDL and NT-proBNP. * *p* < 0.05, ** *p* < 0.01, *** *p* < 0.001 versus low LT-CVR; ^#^ *p* < 0.05, ^###^ *p* < 0.001 versus high LT-CVR.

The low-lifetime CVR group had significantly lower levels of CRP than the high-lifetime CVR or SCAD groups (Figure 1A; *p* < 0.05). Conversely, the high-lifetime CVR group had higher levels of both NT-proBNP (Figure 1B) and oxLDL (Figure 1C) than the low-lifetime CVR (*p* < 0.01 and *p* < 0.001, respectively) and the SCAD (*p* < 0.05 and *p* < 0.001, respectively) groups, and the SCAD group had significantly lower levels of oxLDL than the low-lifetime CVR group (*p* < 0.05). Correlation analysis demonstrated a positive correlation between oxLDL and NT-proBNP in the whole sample (Figure 1D; r = 0.284; *p* = 0.021), whereas CRP showed no correlation with NT-proBNP (r = −0.018; *p* = 0.885) or oxLDL (r = 0.069; *p* = 0.584) (not shown). Multivariate regression analysis showed that the increase in NT-proBNP levels was associated with higher levels of oxLDL independently of LDL, sex, BMI, systolic blood pressure or CRP (Table 2).

### 3.3. Acute Exposure to oxLDL Alters Systolic Ca^2+^ Release in Isolated Rat Cardiomyocytes

**Figure 2:**
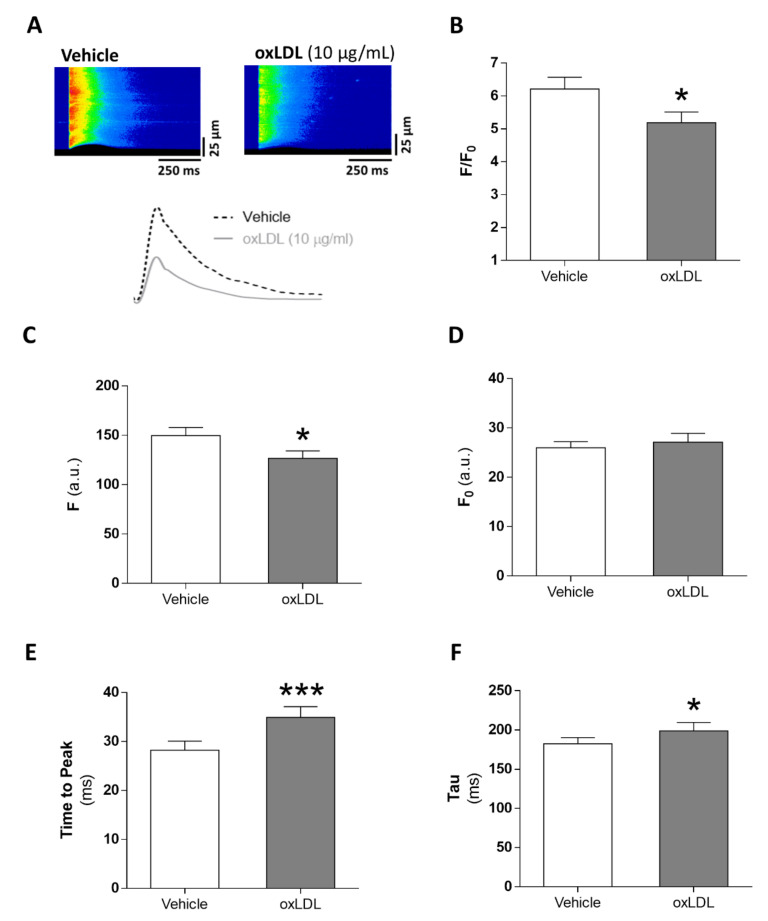
Acute effects of oxLDL on Ca^2+^ transients. (**A**) Representative line-scan images (upper panels) and fluorescent profile (lower panel) of a cardiomyocyte perfused with vehicle (upper left and dotted black line) and after perfusion of 10 µg/mL oxLDL (upper right and continuous grey line) under 1 Hz field stimulation. Mean values of Ca^2+^ transients amplitude (*F/F_0_*, **B**), peak (*F*, **C**) baseline (*F_0_*, **D**), decay time constant (time constant of fitting the descending trace of the fluorescence to an exponential function) (tau, **E**), and time-to-peak (**F**). (n = 32 cells, N = 5 rats). * *p* < 0.05, *** *p* < 0.001 versus vehicle-treated cardiomyocytes.

We next evaluated the acute effects of oxLDL on Ca^2+^ signaling in cardiomyocytes. Representative line-scan images of Ca^2+^ transients recorded in cardiomyocytes after vehicle (left panel) or oxLDL (right panel) perfusion are shown in Figure 2A, along with the superimposed fluorescence profiles (bottom panel). The amplitude of the intracellular Ca^2+^ transients (F/F_0_) was significantly lower in cardiomyocytes acutely exposed to oxLDL than to vehicle (Figure 2B; *p* < 0.05). The decrease in the amplitude was due to a reduction in peak systolic Ca^2+^ transients (F, Figure 2C; *p* < 0.05) and not to changes in the baseline Ca^2+^ fluorescence (F_0_, Figure 2D). Challenge with oxLDL also induced a prolongation in the time-to-peak (Figure 2E; *p* < 0.001) and in the time constant of Ca^2+^ transient decay, Tau (Figure 2F; *p* < 0.05).

### 3.4. Acute Exposure to oxLDL Induces Diastolic SR-Ca^2+^ Leak and a Pro-Arrhythmogenic Profile in the Form of Diastolic Ca^2+^ Waves

**Figure 3:**
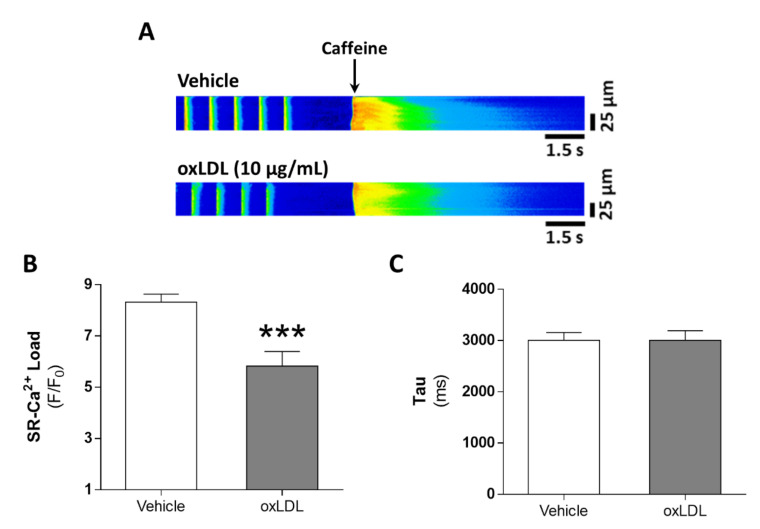
Acute effects of oxLDL on sarcoplasmic reticulum-Ca^2+^ load. (**A**) Representative line-scan images of a cardiomyocyte perfused with vehicle (upper panel) or with 10 µg/mL oxLDL (lower panel) under 1 Hz field stimulation and in the presence of caffeine. Mean values of caffeine-induced Ca^2+^ transients amplitude (*F/F_0_*, **B**) and decay time constant after caffeine perfusion (tau, **C**) in cardiomyocytes treated with vehicle (n = 17 cells, N = 5 rats) or 10 µg/mL oxLDL (n = 20 cells, N = 5 rats). *** *p* < 0.001 versus vehicle-treated cardiomyocytes.

**Figure 4:**
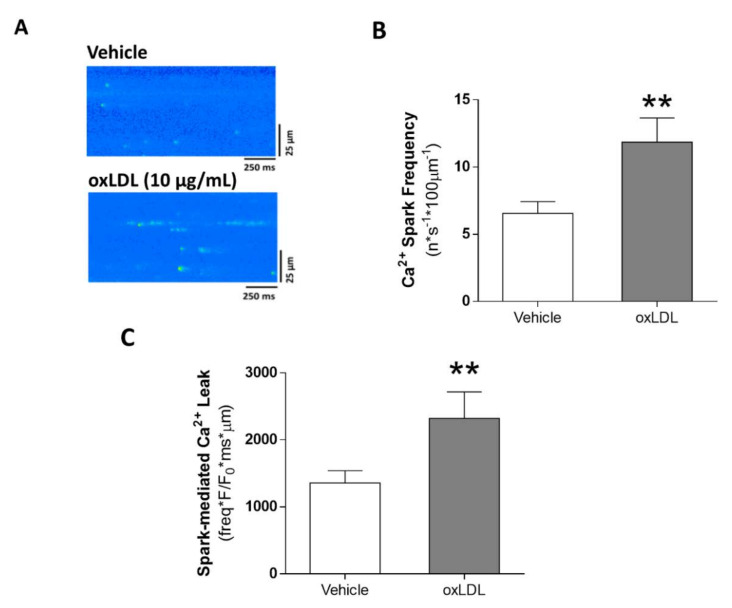
Acute effects of oxLDL on diastolic Ca^2+^ leak. (**A**) Representative line-scan images of Ca^2+^ sparks in a cardiomyocyte perfused with vehicle (upper panel) or 10 µg/mL oxLDL (lower panel). Mean values of Ca^2+^ spark frequency (**B**) and spark-mediated Ca^2+^ leak (**C**) (n = 21 cells, N = 5 rats). ** *p* < 0.01 versus vehicle-treated cardiomyocytes.

Alterations in systolic Ca^2+^ release might be related to alterations in SR-Ca^2+^ load. Therefore, we studied the estimated SR-Ca^2+^ load after caffeine administration. Representative line-scan images of caffeine-induced Ca^2+^ transients in cardiomyocytes challenged with vehicle (upper panel) or oxLDL (bottom panel) are shown in Figure 3A. We found that SR-Ca^2+^ load was significantly reduced following oxLDL perfusion (Figure 3B, *p* < 0.001), but no differences were found in the decay time constant Tau (Figure 3C). The reduced SR-Ca^2+^ load might be due to Ca^2+^ leak from the SR during diastole. Representative line-scan images of Ca^2+^ spark recordings after vehicle (upper panel) or oxLDL (bottom) challenge are shown in Figure 4A. We found that Ca^2+^ spark frequency was significantly higher after oxLDL perfusion than after vehicle perfusion (Figure 4B, *p* < 0.01). Analysis of the biophysical properties of Ca^2+^ sparks revealed that oxLDL induced a reduction in Ca^2+^ spark amplitude (*F/F_0_*), with no differences in duration or width (Table 3). The calculated net spark-mediated SR-Ca^2+^ leak was increased after oxLDL perfusion relative to vehicle (Figure 4C, *p* < 0.01).

**Figure 5:**
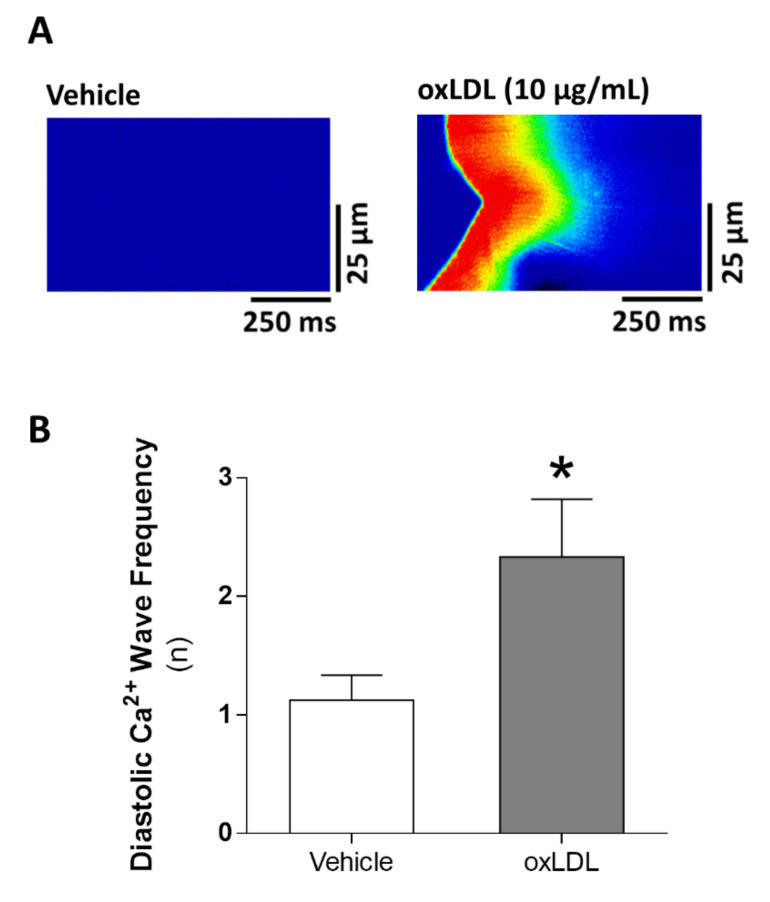
Pro-arrhythmogenic effect of oxLDL in vitro. (**A**) Representative line-scan images of Ca^2+^ waves in a cardiomyocyte perfused with vehicle (left panel) or 10 µg/mL oxLDL (right panel). (**B**) Ca^2+^ wave frequency (n = 21 cells, N = 5 rats). * *p* < 0.05 versus vehicle-treated cardiomyocytes.

Finally, to determine whether oxLDL treatment of intact cells might trigger other forms of pro-arrhythmogenic Ca^2+^ events, we also analyzed the diastolic spontaneous Ca^2+^ release in the form of Ca^2+^ waves after oxLDL challenge in resting quiescent cells, finding a significant increase in the frequency of Ca^2+^ waves (Figure 5A,B; *p* < 0.05).

## 4. Discussion

This study demonstrates, for the first time to our knowledge: (i) an association between oxLDL and NT-proBNP in young adults with or without SCAD and (ii) that acute exposure of adult rat ventricular cardiomyocytes to oxLDL triggers alterations in intracellular Ca^2+^ handling. Our results suggest that an increase in the levels of oxLDL might contribute to ventricular dysfunction in individuals at high CVR before the onset of a coronary event, even from a young age, and that oxLDL predisposes cardiomyocytes to systolic Ca^2+^ release impairment and pro-arrhythmogenic forms of diastolic Ca^2+^ release as a consequence of abnormal intracellular Ca^2+^ cycling. Accordingly, subjects with high levels of oxLDL are at risk of cardiac dysfunction even at a young age.

CVR tends to be estimated on the basis of short-term CVR; however, young adults have low short-term CVR simply because of their young age. Traditional CVR factors such as hypertension, dyslipidemia and obesity have a cumulative impact on CVR which is reflected in lifetime CVR, which is the risk of developing a cardiovascular disease in the remaining lifespan [13,18]. We stratified our population on the basis of lifetime CVR because it is a more representative CVR estimator at young ages. In the young cohort studied here, oxLDL levels increased with lifetime CVR. The exception to this was subjects with SCAD, who had lower levels of oxLDL than peers with low-lifetime CVR despite their higher BMI and the increased prevalence of males and hypertension. While patients with SCAD are considered to be at high CVR, this can be controlled with appropriate pharmacological and lifestyle management [19]. Moreover, our group previously described that the systemic oxidative stress of young subjects with SCAD is similar to that of subjects with low-lifetime CVR [14]. All the subjects with SCAD in our cohort were on statin treatment, which is known to reduce the levels of oxLDL [15]. Therefore, the combination of the lipid-lowering and antioxidant properties of statins [20,21] might contribute to maintaining low levels of oxLDL in well-controlled subjects with SCAD.

In the present study, we describe a similar trend for the levels of the marker of ventricular stress, NT-proBNP, although the levels of NT-proBNP were within the normal range in all groups. NT-proBNP above the normal range is a widely used biomarker of heart failure. Therefore, young subjects who are not susceptible to cardiovascular disease, such as those without SCAD, are not susceptible to high levels of NT-proBNP. Subjects with cardiovascular disease might have high levels of NT-proBNP, as NT-proBNP rises during the acute phase of coronary events and can remain high in the case heart failure development. Subjects in the SCAD group were in the stable phase after the coronary event, did not develop heart failure and were very well-controlled in terms of blood pressure, which could increase volume overload and consequently NT-proBNP. Moreover, all patients with SCAD were on statin treatment, which reduces the levels of NT-proBNP after a cardiovascular event such as acute myocardial infarction [22], and in patients with dilated cardiomyopathy [23] or heart failure [24]. Therefore, it is reasonable that the levels of NT-proBNP were within the normal range in the SCAD group, indicating that NT-proBNP can be normalized after an acute coronary event. In contrast, the higher levels of in NT-proBNP in subjects at high-lifetime CVR, despite remaining within the normal range, indicate that NT-proBNP can be associated with cardiovascular risk even in young subjects not susceptible to cardiovascular disease. In this sense, previous studies described a prognostic value of NT-proBNP in the community [25,26].

The association between NT-proBNP and oxLDL indicates that oxLDL might be directly related to cardiac function both before and after the cardiovascular disease. oxLDL has been associated with a decrease in cardiac function in the general population [27] and in patients with congestive heart failure [28]. This association might, however, be confounded by traditional CVR factors, which are typically associated with oxidative stress. Moreover, LDL has a direct impact on the levels of oxLDL, and hypertension increases volume overload and consequently the levels of NT-proBNP. We thus performed multivariate regression analysis to assess whether NT-proBNP levels are affected by oxLDL independently of LDL, systolic blood pressure and the traditional CVR factors showing differences between groups. We found that the levels of NT-proBNP increased with oxLDL and systolic blood pressure, which were mutually independent. Additionally, although the levels of LDL could affect the levels of oxLDL, the association between NT-proBNP and oxLDL was independent of LDL and the remaining traditional CVR factors. In fact, the levels of NT-proBNP were not associated with the levels of LDL.

A pro-inflammatory state is known to increase the levels of oxLDL, which triggers the activation of pro-inflammatory pathways in a positive feedback loop [2]. We thus assessed the levels of CRP as a major marker of systemic inflammation, finding a lack of association with both oxLDL and NT-proBNP. Although CRP follows the same trend as oxLDL and NT-proBNP in subjects without SCAD, it remains high in subjects with SCAD. CRP is known to dramatically increase during the acute phase of coronary events, and statins are known to reduce the levels of CRP [29]. Treatment with statins in subjects with SCAD might have reduced the levels of CRP after the acute phase of the coronary event to levels of the high-lifetime CVR group. Unlike oxLDL, CRP is not a specific marker of cardiovascular disease, and is not currently considered a target for treatment. Therefore, although CRP levels might have been affected by statins, it was not as well-controlled as oxLDL or NT-proBNP. In this sense, while high BNP levels are associated with a worse prognosis in the general population [25] and in individuals with SCAD [30] or with a history of heart failure [26,31], the data on the association between CRP and cardiovascular disease are conflicting [32,33,34], likely because of the dependence of CRP on traditional CVR factors [35]. Therefore, multivariate regression analysis also demonstrated that the association between NT-proBNP and oxLDL was independent of CRP levels. Our results are in agreement with previous work showing a lack of association between oxLDL and CRP in subjects with an acute coronary event, suggesting the involvement of independent molecular pathways [3]. Accordingly, NT-proBNP is a stronger indicator of cardiac dysfunction than CRP in the general population [26] and in subjects with SCAD [36] or heart failure [37].

Related to the cellular effect of oxLDL, authors indicated that oxLDL induces apoptosis in neonatal rat cardiomyocytes [9,38,39] and increases the expression of BNP in the HL-1 atrial cell line [40]. NT-proBNP predicts first-onset cardiac arrhythmia and sudden cardiac death in patients [26,30], and also reflects malignant electrophysiological remodeling [32]. Both oxLDL and BNP are independent predictors of response to cardiac resynchronization in heart failure, and are reduced in response to therapy [41], but much less is known about the role of oxLDL in cardiac events such as ventricular arrhythmias. We therefore analyzed the direct effect of oxLDL on Ca^2+^ handling and in the pro-arrhythmic Ca^2+^ release during diastole in adult rat ventricular cardiomyocytes. Acute exposure to oxLDL induced alterations in Ca^2+^ handling that are similar to those found in human heart failure, such as compromised systolic Ca^2+^ release [7,11,42]. Rapid elevations of oxLDL at the systemic level could be frequent in cardiovascular disease and, in this context, our findings of a reduction in systolic Ca^2+^ release and SR-Ca^2+^ load and an increase in spontaneous SR-Ca^2+^ release in the form of Ca^2+^ sparks and waves frequency indicate that oxLDL could trigger pro-arrhythmogenicity in adult ventricular cardiomyocytes. In support of this, treatment of guinea pig adult cardiomyocytes with oxLDL induced an increase in pro-arrhythmogenic activity [43]. Therefore, our results suggest that oxLDL induces alterations in intracellular Ca^2+^ cycling in ventricular cardiomyoyctes.

The effects of oxLDL on SR-Ca^2+^ leak in the form of spontaneous Ca^2+^ sparks and waves suggest that it can directly induce a pro-arrhythmogenic profile. The increased decay time of Ca^2+^ transients after challenge with oxLDL indicates that SERCA pump activity is impaired, and therefore intracellular Ca^2+^ uptake to the SR might be also altered. SR-Ca^2+^ load is decreased both because of the worsened intracellular Ca^2+^ uptake and because of the increase in spontaneous SR-Ca^2+^ leak. The decay time of Ca^2+^ transients induced by caffeine, which represents the activity of NCX, is not affected by oxLDL. Supporting these data, a previous study failed to show alterations in NCX after treatment of guinea pig adult cardiomyocytes with oxLDL [43].

Serum lipids are suggested to affect cardiac function independently of their effect on the vascular system through multiple mechanisms including electrical cardiac remodeling [44]. However, the controlling mechanisms are currently unknown. Ventricular arrhythmia often leads to sudden cardiac death, mainly in the convalescent phase after myocardial infarction [45,46], and is suggested to be caused by a cascade of events triggered by ischemia/reperfusion [47]. In this regard, LDL predicts short-term risk of sudden cardiac death in asymptomatic subjects [46] and in patients with SCAD [48,49], and is associated with ventricular tachyarrhythmia and fibrillation in patients with acute myocardial infarction [50]. Studies on animal models of hypercholesterolemia have also described alterations in Ca^2+^ handling [51,52,53]. In fact, hyperlipidemic *ApoE*-deficient mice have a higher risk of arrhythmia post-myocardial infarction than wild-type mice [54]. Accordingly, statins exert antiarrhythmic effects suggested to be associated with their antioxidant and anti-inflammatory effects [55] that, together with lipid-lowering effects, reduce oxLDL. Therefore, the burst in ROS production caused by ischemia/reperfusion might induce LDL oxidation at the myocardium, contributing, at least in part, to subsequent arrhythmia development.

This study has some limitations that should be addressed. First, the proportion of male subjects is significantly higher in the high-lifetime CVR and the SCAD groups than in the low lifetime CVR group. Women have better cardiovascular risk profiles than men prior to menopause, presenting lower blood pressure and LDL, and higher HDL, than their male peers [56]. Therefore, CVR estimators confer higher levels of CVR to male subjects than to their female peers. The age range of our population was 30–50 years, so it is reasonable that a higher proportion of females was located in the low-lifetime CVR group, while a higher proportion of males was located in the high-lifetime CVR group. Similarly, the prevalence of male subjects among subjects with history of cardiovascular disease is greater than the prevalence of female peers. However, in this study, multivariate regression analysis clearly confirmed that the association between oxLDL and NT-proBNP was independent of sex. Second, additional data on cardiac function or concomitant cardiovascular drugs might further explain the trend followed by NT-proBNP and oxLDL. The population described in this study was recruited during the annual employee health checkup, and data associated with secondary care such as echocardiography were not collected. However, Rietzchel et al. previously described an association between oxLDL and echocardiographic parameters of cardiac function in the general population [27]. Third, the in vitro study is only descriptive of the oxLDL-induced effect in adult ventricular cardiomyocytes. Therefore, further studies are needed to unravel the molecular pathway triggered by oxLDL. Particularly, antioxidant pretreatments might rescue the oxLDL-induced impairment of Ca^2+^ handling given the well-known pro-oxidant effect of oxLDL.

## 5. Conclusions

In conclusion, the present work demonstrates a direct association between ventricular stress and oxLDL in young adults with and without previous coronary events, and also a direct functional effect of oxLDL on the regulation of Ca^2+^ dynamics that may contribute to arrhythmia development. Young subjects tend to underestimate their CVR because of their low short-term CVR, which can lead to poor control of CVR factors. Our results show the importance of controlling the levels of oxLDL even before the onset of the coronary event in order to prevent cardiac dysfunction and arrhythmia development.

## Figures and Tables

**Table 1:** Baseline characteristics of the study population.

	Low-Lifetime CVR (*n* = 22)	High-Lifetime CVR (*n* = 21)	SCAD (*n* = 23)
Age (years)	44.1 ± 4.0	43.9 ± 5.3	44.8 ± 4.4
Males (n, %)	6 (27)	16 (76) **	19 (83) ***
BMI (kg/m^2^)	23.4 ± 2.6	29.8 ± 4.4 ***	28.3 ± 5.7 **
Hypertension (n, %)	0 (0)	13 (62) ***	9 (39) **
SPB (mmHg)	111.7 ± 9.1	137.3 ± 12.3 ***	122.8 ± 20.0 *^,##^
DBP (mmHg)	71.1 ± 9.9	88.3 ± 9.8 ***	76.3 ± 12.3 ^###^
Antihypertensive treatment (n, %)	0 (0)	8 (38) **	8 (35) **
Dyslipidemia (n, %)	0 (0)	9 (43) ***	3 (13) ^#^
Total cholesterol (mg/dL)	188.1 ± 29.7	210.7 ± 38.9 *	143.5 ± 41.6 ***^,###^
LDL-cholesterol (mg/dL)	99.4 ± 23.2	137.2 ± 35.8 ***	78.5 ± 39.4 *^,###^
HDL-cholesterol (mg/dL)	72.7 ± 16.7	41.2 ± 11.5 ***	39.3 ± 9.5 ***
Triglycerides (mg/dL)	75.5 ± 28.4	207.8 ± 100.8 ***	116.2 ± 56.4 ^###^
Statin treatment (n, %)	0 (0)	5 (24) *	23 (100) ***^,###^
Renal function			
eGFR (mL/min/1.73m^2^)	98.0 ± 11.0	96.5 ± 11.0	97.8 ± 16.3
Serum creatinine (mg/dL)	0.797 ± 0.115	0.905 ± 0.136	0.892 ± 0.191
Cardiovascular risk			
QRisk-lifetime (%)	21.3 ± 2.7	42.9 ± 9.2 ***	

CVR = cardiovascular risk, SCAD = stable coronary artery disease, BMI = body mass index, SBP = systolic blood pressure, DBP = diastolic blood pressure, LDL = low-density lipoprotein, HDL = high-density lipoprotein, eGFR = estimated glomerular filtration rate. * *p* < 0.05, ** *p* < 0.01, *** *p* < 0.001 versus low-lifetime CVR; ^#^ *p* < 0.05, ^##^ *p* < 0.01, ^###^ *p* < 0.001 versus high-lifetime CVR.

**Table 2:** Multivariate regression analysis of N-terminal pro-B-type natriuretic peptide, oxidized low-density lipoprotein, traditional cardiovascular risk factors and C-reactive protein.

	*B*	CI	*p*-Value
oxLDL	0.459	0.11–0.81	0.011
LDL	−0.078	−0.21–0.05	0.227
Sex	7.223	−1.21–15.66	0.092
BMI	−0.831	−1.80–0.14	0.092
SBP	0.353	−0.097–0.61	0.008
CRP	−0.313	−2.39–1.76	0.763

oxLDL = oxidized low-density lipoprotein, CRP = C-reactive protein, CI = confidence interval, BMI = body mass index, SBP = systolic blood pressure.

**Table 3:** Ca^2+^ spark characteristics in cardiomyocytes acutely perfused with vehicle or 10 µg/mL oxidized low-density lipoprotein.

Treatment	Peak (F/F_0_)	Full Width at Half Maximum (µm)	Full Duration at Half Maximum (ms)	N (Sparks/Cells/Rats)
**Vehicle**	1.75 ± 0.18	3.73 ± 0.57	29.5 ± 4.7	1014/21/5
**oxLDL**	1.06 ± 0.16 *	3.57 ± 0.46	31.6 ± 8.1	1325/21/5

oxLDL = oxidized low-density lipoprotein, * *p* < 0.05 versus vehicle-treated cardiomyocytes.

## Abbreviations

BMI	body mass index
CRP	C-reactive protein
CVR	cardiovascular risk
EC	excitation–contraction
LDL	low-density lipoprotein
NCX	Na^+^-Ca^2+^ exchanger
NT-proBNP	N-terminal pro-B-type natriuretic peptide
oxLDL	oxidized LDL
ROS	reactive oxygen species
SCAD	stable coronary artery disease
SERCA	sarcoplasmic reticulum Ca^2+^-adenosine triphosphatase 2a
SR	sarcoplasmic reticulum
